# *Candida* prevalence and oral hygiene due to orthodontic therapy with conventional brackets

**DOI:** 10.1186/s12903-020-01267-4

**Published:** 2020-10-10

**Authors:** Kinga Grzegocka, Paweł Krzyściak, Anna Hille-Padalis, Jolanta E. Loster, Katarzyna Talaga-Ćwiertnia, Bartłomiej W. Loster

**Affiliations:** 1 Department of Orthodontics, Dental Institute, Faculty of Medicine, Jagiellonian University Medical College, ul. Montelupich 4/108, 31-155 Kraków, Poland; 2 Department of Mycology, Chair of Microbiology, Faculty of Medicine, Jagiellonian University Medical College, Kraków, Poland; 3 Department of Prosthodontics, Dental Institute, Faculty of Medicine, Jagiellonian University Medical College, Kraków, Poland

**Keywords:** Orthodontic brackets, *Candida*, Oral hygiene, Periodontal index

## Abstract

**Background:**

Conventional brackets are often used during orthodontic therapy of patients with malocclusion. The complex construction of such brackets greatly inhibits oral hygiene, which predisposes to increased carriage of microbiota. Orthodontic brackets could act as reservoir of yeast and predispose to oral candidosis. The aim of this study was to assess *Candida* prevalence and the role of oral hygiene during fixed appliance therapy*.* A further aim was to characterize the isolated yeasts according to their ability to form biofilms.

**Methods:**

Seventeen participants (average age 17 ± 7 years) were monitored by taking oral rinses and elastomeric ligature samples, and by evaluating the approximal plaque index (API) and gingival bleeding index (GBI) before and after placement of the orthodontic conventional brackets for 12 weeks. Isolated yeasts were counted and biofilm formation was evaluated.

**Results:**

One hundred and sixteen samples (67 oral rinses and 49 orthodontic elastomers) were collected. Ten patients (58.8% subjects) were *Candida-*carriers (two were colonized after bracket placement) and *C. albicans* was the most common species. The average number of yeasts in the oral cavity showed some fluctuation during the study, but in general had an upward trend (adj. R2 = 0.7967, *p* = 0.07025). A correlation was found between median number of yeasts and the periodontal indices (API, GBI). The average API values decreased in the *Candida*-carriers (adj. *R*^2^ = 0.95; *p* = 0.01709), while average GBI values increased in the noncarriers (adj. *R*^2^ = 0.92; *p* = 0.0256).

**Conclusions:**

Treatment with orthodontic appliances promotes *Candida* yeast colonization, which is variable over time in terms of strain and species, with dominance of *C. albicans*, and without increased biofilm-forming activity. The API value decreases over time in carriers, and the GBI value increases in uncolonized patients, which may have predictive significance for the development of oral candidiasis during orthodontic treatment.

## Background

Conventional brackets are often used during orthodontic therapy of patients with malocclusion. The complex construction of such brackets greatly inhibits oral hygiene, which predisposes to increased carriage of bacteria and yeasts [1, 2]. An increased amount of fungi in the oral cavity can, with predisposing factors, lead to the development of oral mycosis. A count of yeast colonies (colony-forming unit, CFU) can be used to estimate the number of fungi, calculated from the volume of washings collected from the patient’s mouth. This method allows both the number composition (quantitative assessment) and species composition (qualitative assessment) of isolated colonies to be determined, unlike swabs, which are less reliable for technical reasons (they require sampling from a specific surface area (e.g., 1 cm^2^) or the same location (such as the palate; they also involve a high risk of contamination through touching other anatomical structures, and are prone to errors related to adherence and recovery of colonies from a swab) [3]. The state of oral hygiene can also be expressed numerically, as with the periodontological indices. One of the most popular dental indicators is the approximal plaque index (API), which expresses the amount of uncleaned interdental space as a percentage. API values of 25–39% indicate good hygiene, and < 25% point to optimal hygiene. A permanent lack of good oral hygiene causes inflammation of the gums, the first symptom of which is bleeding. The gingival bleeding index (GBI) is used to assess gum inflammation. GBI values of below 10% indicate clinically healthy periodontium.

One element of conventional orthodontic brackets are elastomeric rings, which are used to connect the bracket with the orthodontic wires. The irregularity and roughness of the surface of these rings are favorable to colonization and biofilm formation by microorganisms [4, 5]. Biofilms are usually multispecies assemblages of microorganisms encased in a matrix a mode of life common to most microorganisms in natural and medical systems (as well as components of orthodontic appliances), which allows survival in hostile environments. A biofilm can become a reservoir of pathogens and, with predisposing factors, can contribute to thrush and other forms of oropharyngeal candidosis. One factor in Candida’s virulence is the ability of yeasts to form biofilms, and it would seem that strains isolated from patients colonized and using orthodontic appliances will form a biofilm well [6, 7].

Even though *Candida* is a part of the normal oral microbiota, being found in 17–75% human population, it can often be the cause of oral mycoses, especially in immunodeficient patients [8, 9]. The most common etiological factor of oral candidosis is *Candida albicans.* However, *C. tropicalis, C. glabrata, C. parapsilosis, C. krusei* sometimes occur with high prevalence, especially in susceptible patients such as diabetics [10–12]. All these species have a great ability to form biofilms, especially with oral gram positive bacteria [8–11].

Very few studies have compared *Candida* prevalence and *Candida* growth in orthodontic patients before, during, and after treatment [1, 12]. The small number of papers related to this topic and the correlation between orthodontic elastomeric rings and oral *Candida* growth led to this study.

The purpose of this study was to investigate the occurrence of *Candida* species and the role of oral hygiene, as measured by periodontal parameters during fixed appliance therapy. A further aim was to characterize the isolated *Candida* species by their ability to form biofilms, which are a virulence factor in the development of candidosis related to orthodontic therapy.

## Methods

### Patients and samples

Seventeen patients (eleven females and six males, aged 11–30 years old, average age 17 ± 7 years, median 14 years) at the Department of Orthodontics, University Dental Clinic, Kraków, Poland, were randomly selected. Due to occlusion defects, all subjects required orthodontic treatment using conventional brackets and gave their written consent to participate in the study. The inclusion criteria were healthy individuals, both sexes, aged ≤30 years old. The exclusion criteria were oral mucosa disease; smoking; use of antibiotics, corticosteroids, or any hormone medication in the 3 months prior to the study; pregnancy, or breastfeeding. All patients were informed that the use of antimicrobial mouthwashes was prohibited during the study.

The research project included a schedule of four visits: T0: before bonding brackets; T1, T2, and T3: approximately 2, 6, and 12 weeks after bonding brackets, respectively.

During the study, patients used the same type elastic rings (color Glow Blue, catalogue index OCLGB, Orthodontic Design and Production, USA), metal brackets Cannon Ultra System (Orthodontic Design and Production, USA), and NiTi wires (Fairfield Orthodontics, USA).

All participants were thoroughly instructed by one of the authors on how to properly take care of oral hygiene during orthodontic treatment. Patients reported for all appointments properly prepared (in accordance with written recommendations): in the morning, on an empty stomach (minimum 6 h after eating), before morning toothbrushing and other oral hygiene procedures. Patients were also asked to limit exercise and follow a mixed diet on the day preceding the study.

During visit T0, before brackets were bonding, oral hygiene was assessed using the approximal plaque index (API), following Lange, and the gingival bleeding index (GBI), following Ainamo and Bay. Oral rinses were collected.

To measure API, a periodontal probe was gently guided through the approximal spaces of the first and third quadrants from the oral aspect, and of the second and fourth quadrants from the buccal aspect. The presence of plaque deposits was recorded as a positive result. The percentage of sites with positive results was counted.

The GBI was taken by gently probing the orifice of the gingival crevice. If bleeding occurred within 10 s, a positive finding was recorded. The number of positive sites was recorded and then expressed as a percentage of the number of sites examined.

Collection of mycological material was performed using oral rinses. Patients under the supervision of the orthodontist rinsed their mouth for 60 s with 10 ml isotonic saline (0.9% NaCl) at room temperature, and spat out the rinses into a sterile container, which was immediately delivered to the mycological laboratory.

During visits T1, T2, and T3, the API and GBI indices were re-evaluated, and oral rinses and elastomeric rings were collected. Orthodontic ligatures were placed into Eppendorf tubes filled with saline solution. Elastomers were collected using a sterile dental kit in aseptic conditions to prevent material contamination.

### Microbiological analysis

#### Total candida and mean candida carriage

The mouth washing samples were vigorously shaken for 90 s with a Vortex shaker, and then quantitatively plated on Sabouraud’s chloramphenicol agar (Biocorp) and incubated at 35 ± 2 °C for 72 h. The collected elastomeric ligatures were inoculated directly onto the medium. The grown colonies were identified based on classical mycological methods, such as colony morphology, the Dalmau plate technique, and an API 20C AUX commercial assimilation test (Biomerieux).

Strains were collected and frozen for further biofilm formation studies.

#### In vivo biofilm scanning electron microscopy (SEM)

Several randomly selected ligatures, present in the oral cavity for about 4 weeks, with known *Candida* growth were subjected to a scanning electron microscope analysis to assess the biofilm produced in vivo. The topography of an unused elastic ligature was also examined as a control. Analysis of the prepared samples was performed using a JEOL JSM-35CF scanning microscope (JSM-35CF; JEOL Vacuum Evaporator) at the Laboratory of the Otolaryngology Clinic, University Hospital, Kraków.

#### Candida biofilm formation

The biofilm formation assay was performed as follows: the overnight culture of investigated strains was transferred to sterile saline, and the fungal suspension was adjusted to 1 on McFarland scale with a densitometer (DEN1 Biosan, Lithuania). One hundred μL of standardized suspensions (eight wells per strain) were added to each well of sterile 96-well flat-bottom polystyrene plates filled previously (100 μL per well) with double concentrated Roswell Park Memorial Institute (RPMI) 1640 medium with L-glutamine without bicarbonate (Sigma Aldrich), supplemented with 2% glucose (Avantor Performance Materials, Gliwice, Poland) and buffered with 3-morpholinopropane-1-sulfonic acid (MOPS, Sigma Aldrich). The plates were incubated for 1.5 h at 37 °C for the adherence phase, before being washed twice with sterile phosphate buffered saline (PBS) to remove nonadherent cells. Each well was then filled with new RPMI medium and incubated without shaking for 72 h at 37 °C. After that time, the plates were washed with PBS, dried in air for 45 min, and stained with 125 μL per well of 0.1% crystal violet solution (Avantor Performance Materials, Gliwice, Poland) for 45 min at room temperature. The microtiter dish was then washed and then dried. A 150 μL volume of 95% ethanol (Avantor Performance Materials, Gliwice, Poland) was added to each well, and then plates were covered and incubated for 45 min at room temperature. A 100 μL sample of the resulting ethanol-crystal violet solution was then transferred from each well to a new microtiter plate, and the optical density (OD) was determined at a wavelength of 570 nm (Infinite 200 Pro Tecan Männedorf, Switzerland). The experiment was repeated, with each strain being analyzed in sixteen replicates.

The study included 27 isolates from ten patients and one reference strain of *C. albicans* (ATCC 90028).

### Statistical analysis

To detect differences in GBI, API, and number of *Candida* colony-forming unit (CFU) across multiple tests, we used one-way repeated measures analysis of variance by ranks (Friedman test and Skillings–Mack test). Differences in biofilm formation among *Candida* strains were evaluated using the Kruskal–Wallis test with Dunn’s post hoc analysis. All statistical analysis was carried out using R software [13, 14], and a *p*-value below 0.05 was considered significant.

## Results

### Candida carriage

One hundred and sixteen samples were collected from seventeen patients (67 oral rinses and 49 orthodontic ligatures samples). Positive *Candida* growth was noted in 52 samples (34 oral rinses comprising 51% of samples, and 18 orthodontic ligatures samples comprising 37%) and *C. albicans* was the most commonly isolated species (91.1%), followed by *C. tropicalis* (4.5%) and *C. guilliermondii* (4.5%).

**Fig. 1:**
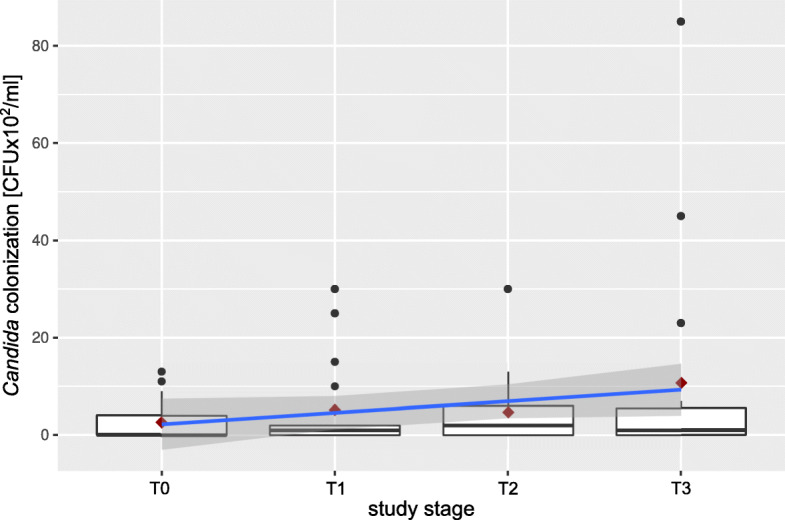
Occurrence of *Candida* in the oral cavity of *Candida* carriers throughout the study (shown as a boxplot with average number of colonies). The blue line connecting the averages ( ) shows an upward trend; the grey area is the confidence interval for the mean

Analysis of *Candida* carriage among oral rinses before bonding brackets shown that eight patients (47%) had *Candida* with an average number of 5.5 × 10^2^ ± 4.8 × 10^2^ CFU/ml. Two further patients (11.8%) were colonized during the study. In total, ten patients (58.8%) were *Candida*-carriers, of which 6 (35%) were consistent carriers (positive results throughout the whole study) and four (23.5%) were inconsistent carriers (negative result at least once in the study). The average number of yeast colonies fluctuated over the study, with little decline at stage T2. At stages T0, T1, T2, and T3, the *Candida*-carriers had 4.4 × 10^2^, 8.8 × 10^2^, 8 × 10^2^, 190 × 10^2^ CFU/ml, respectively (Table 1). The highest CFU value of 8.5 × 10^3^ CFU/ml was found in one patient (P.M.) in stage T3. The average number of colonies per unit volume obtained from oral rinses showed an upward trend depending on the study stage (adjusted *R*^2^ = 0.7967), but without statistical significance (*p* = 0.07025) (Fig. 1).

**Table 1:** Average value of *Candida* sp. (CFU/ml) and API and GBI indices (%) during the study for all patients

		T0	T1	T2	T3	Differences between stages
*Candida* colonization [CFU/ml count]	Average ± SD (range) Median	2.6 × 10^2^ ± 4.3 × 10^2^ (0–1.3 × 10^3^) 0 × 10^2^	5.2 × 10^2^ ± 9.4 × 10^2^ (0–3.0 × 10^3^) 1 × 10^2^	4.7 × 10^2^ ± 7.6 × 10^2^ (0–3.0 × 10^3^) 2 × 10^2^	10.7 × 10^2^ ± 23.1 × 10^2^ (0–8.5 × 10^3^) 1 × 10^2^	*p* = 0.9092 (Skillings–Mack Statistic)
API value [%]	Average ± SD (range) Median	40.9 ± 22 (13–94.8) 42.3	43.4 ± 22.6 (13.6–88.2) 38.0	38.3 ± 17.4 (10–66.7) 35.3	39.8 ± 23 (4.5–100) 35.0	*p* = 0.7968 (Friedman rank sum test; chi-squared)
GBI value [%]	Average ± SD (range) Median	10.3 ± 9 (0–25) 8.3	9.4 ± 8.8 (0–31.2) 7.5	19.23 ± 24.8 (0–93.8) 11.8	10.6 ± 8.5 (0–29.5) 12.5	*p* = 0.4929 (Friedman rank sum test; chi-squared)

Legend: *API* approximal plaque index, *GBI* gingival bleeding index, *SD* standard deviation, *CFU* colony-forming unit

### API and GBI results

The mean API and GBI values at stage T0 were 41% ± 22 and 10% ± 9, respectively. There were no differences in the distribution of GBI values for all patients between study stages (Friedman rank sum test chi-squared = 2.4041, df = 3, *p*-value = 0.4929) or differences in API values (Friedman rank sum test; chi-squared = 1.0185, df = 3, *p*-value = 0.7968).

**Fig. 2:**
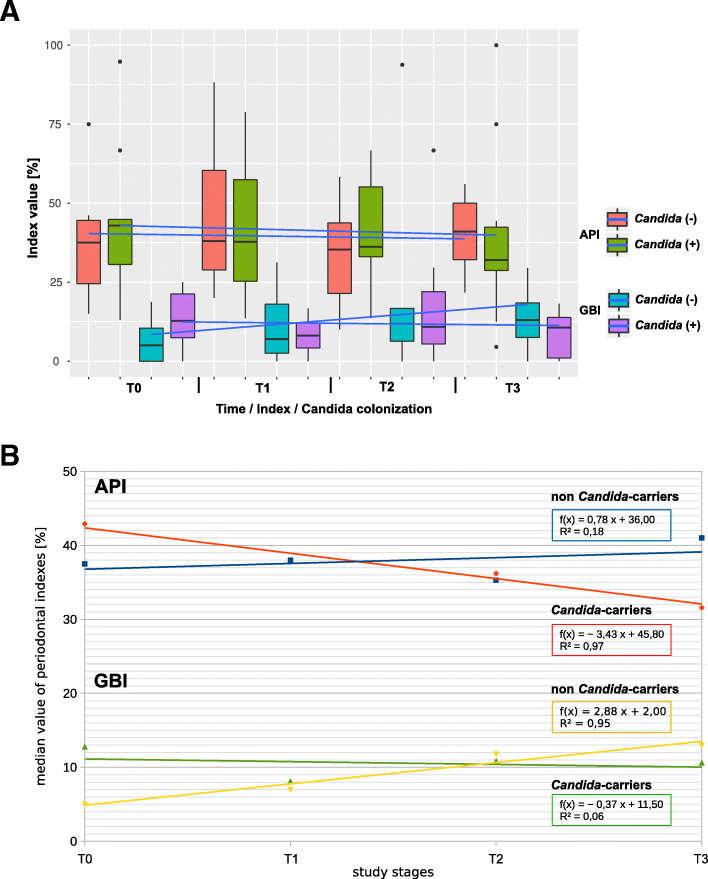
**a** Changes in API and GBI indices on the duration of the study among *Candida* positive and *Candida* negative patients. Blue line connects the average value. **b** The same correlation as in Fig. 2, but showing the correlation with median value among *Candida* positive and *Candida* negative patients

When the study groups were divided into two subgroups: (1) non-*Candida*-carriers, comprising 41%, and (2) *Candida*-carriers (patients in whom yeast growth was found at any stage of sampling), comprising 59%, some tendencies for changes in median index values were observed. In *Candida*-carriers, medians of API values decreased (adjusted R-squared coefficient = 0.94; *p* = 0.01709), while in non-*Candida*-carriers, the medians of GBI values increased (adjusted R-squared coefficient = 0.92; *p* = 0.0256) (Fig. 2a, b).

### Biofilm formation: SEM results

**Fig. 3:**
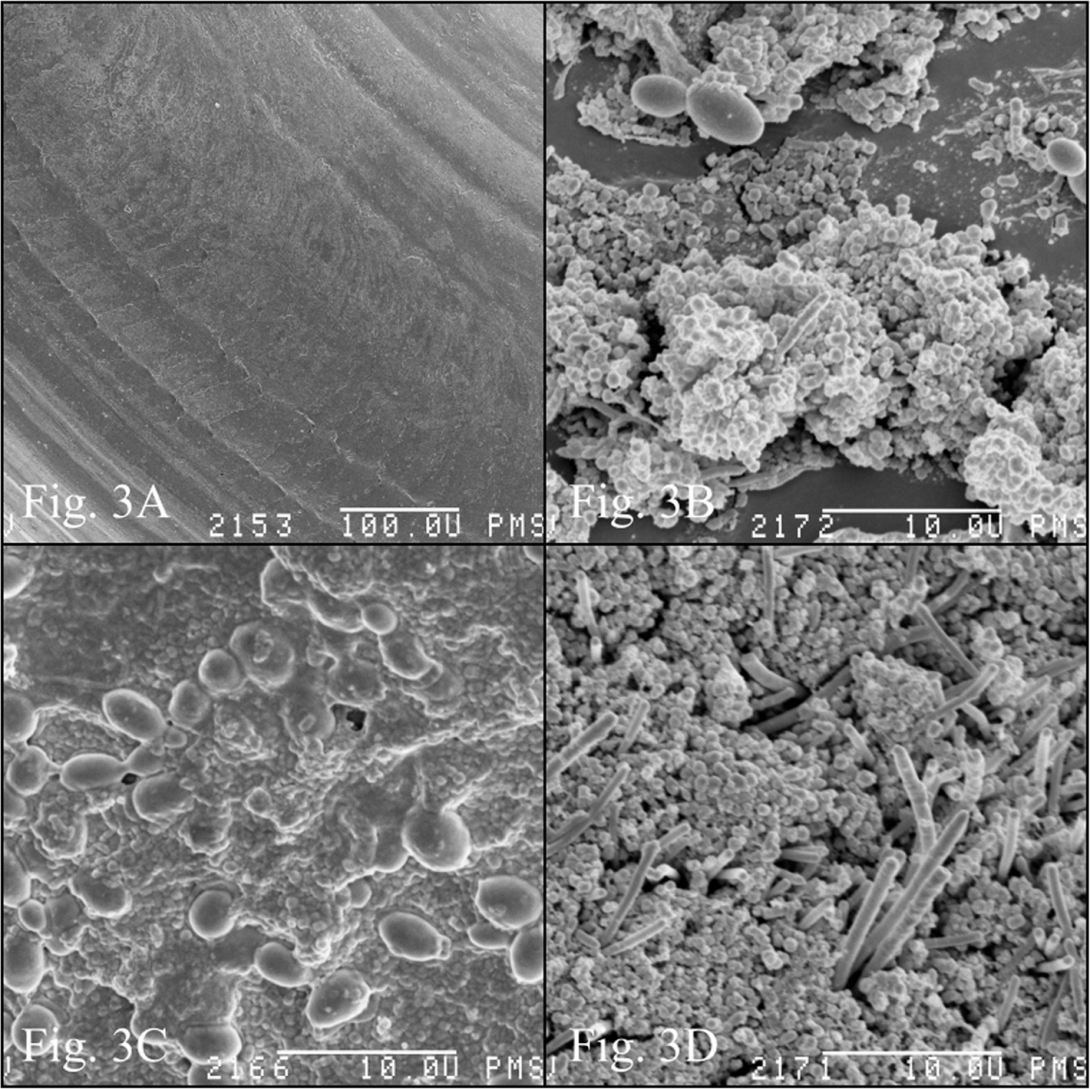
**a** The electron microscopy image of clear orthodontic ligatures: no microbial contamination. **b, c, d** Orthodontic ligatures topography after four weeks’ in the oral cavity. **b** Budding yeast cells. **c** Yeast cells in dense bacterial biofilm. **d** Bacterial biofilm, with rods rising straight from cocci layer

Topographic assessment of unused elastic rings under an electron microscope showed a clean surface, free of microorganisms (Fig. 3a).

On the surface of the colonized orthodontic elastic ligatures, microorganisms created a multicellular, architecturally complex structure, with the presence of various types of bacteria (cocci, bacilli, rods) and yeast (both early and late stage of biofilm formation) (Fig. 3b, c, d).

### Ability to form biofilm

**Fig. 4:**
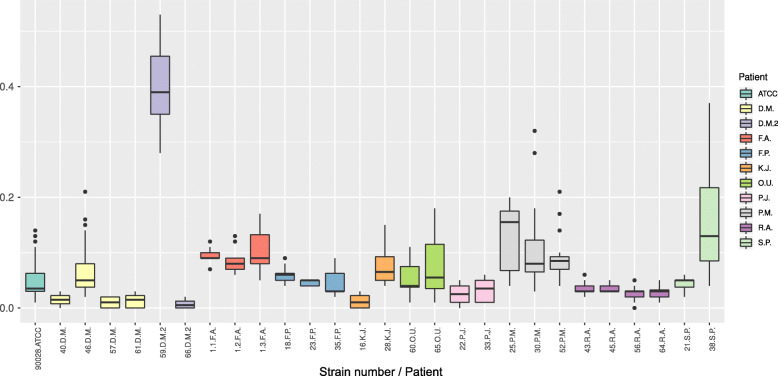
Ability to form biofilm of isolated strains with comparison to reference *C. albicans* strains (ATCC 90028)

Most of the examined strains created biofilm (Fig. 4). Pairwise comparisons with control [14] shows the difference in biofilm production for seven strains (Dunn’s post hoc test after Kruskal–Wallis test; *p* < 0.05). These strains (59, 28, 65, 25, 30, 52, 38) produced significantly more biofilm biomass than *Candida albicans* ATCC 90028.

In the overwhelming majority of cases, biofilm formation was homogenous among strains isolated from particular patients (F.A., F.P., O.U., P.J., P. M., R.A., and S.P.). In only few cases, when various species were isolated (i.e., patient D.M.2, and K.J.) and in one case of *Candida albicans* isolation (D.M.), the biofilm formation differed by study stage.

There is no correlation between biofilm forming ability and API or GBI index (adjusted *R*^2^ = 0.02, *p* = 0.2377, adj. *R*^2^ = 0.04, *p* = 0.1824, respectively).

## Discussion

In the current study, the percentage of *Candida*-carriers before orthodontic treatment was relatively high, at 47% of patients. It increased during orthodontic treatment to almost 59% (nearly 12% of subjects became carriers). This would not be expected given the occurrence of *Candida* in the Polish population, affecting an average of 30.6% of healthy individuals [15]. However, the study citied used oral swabs (*N* = 654; 7–45 years old) and the lower result may be the effect of the different collecting method. Tooyama et al. [3] showed that the test result depends significantly on the sampling method. In his study of Japanese patients (*N* = 200, average age 47.2 years old), when measured using oral swabs, *Candida* affected 33.5% of patients, whereas 52% of the same group of patients were found to be colonized with *Candida* sp. when concentrated rinse was used as the collecting method, comparable with our results and methodology. This shows that the method of sampling has a great impact on the results, and that not all studies can be directly compared.

Zheng et al. [16] determined that oral *Candida* sp*.* was present in only 14% of young Chinese adults (*N* = 50, average age 13.6 years old) before the application of fixed orthodontic appliance. This lower result may be the effect of toothbrushing prior to sampling. Arslan et al. [17] tested a Turkish population (*N* = 72, average age 19.6 years old) using saliva samples and oral swabs, finding high *Candida*-carriage rate of 58.5%. This may suggest the occurrence of large population variables, both within age and ethnic groups, as carriage of fungi depends on many factors, such as diet and lifestyle.

In our investigation, two patients (nearly 12%) became *Candida-*carriers over the course of the experimental phase of the study (after bracket placement). This is coherent with other publications that have suggested that treatment with orthodontic appliances promotes *Candida* yeast colonization. Hägg et al. [18] demonstrated using the imprint technique that overall *Candida* prevalence increases after bonding brackets (*N* = 27, mean age 15 years old).

Lee et al. [19] described that 15% of subjects became *Candida*-carriers in their study using oral concentrated rinse analysis of Chinese patients (*N* = 112, average age 17.7 years old), amount to an increase in *Candida*-carriers from an initial 32% (T0: before bracket bonding) to a maximum of 50% (T5: around 5 months after bonding). They reported that 11% of subjects were consistent carriers of *Candida* species, 64% were inconsistent carriers, and only 25% were consistent non*-*carriers (i.e., never carried *Candida* species throughout the experimental period of 12 months)*.* We were also able to distinguish a group of consistent (35%) and inconsistent (23.5%) *Candida*-carriers. This may indicate that the initially tested noncarrier group at the study baseline contained some false negative results. It needs to be considered that the introduction of orthodontic appliance is only one variable factor influencing the prevalence of *Candida* yeast in the oral cavity (others being diet, oral hygiene, lifestyle, immune system, etc.). In future studies, longer observation of the study group with multiple tests is recommended to isolate the group of consistent and inconsistent carriers before the start of the experimental phase of the study.

In our study, a quantitative analysis of *Candida* colonies in carriers group was carried out, as the number of yeast colonies indicates development of oral yeast infection during orthodontic treatment. Tooyama et al. [3] determined the reference ranges of *Candida* colonies for healthy commensal carriages at 0–5 CFU/swab and 0–670 CFU/ml with the concentrated rinse method. In our study, five patients had an average colony count higher than 670 CFU/ml, but clinically did not show any symptoms suggesting that *Candida* grew on the abiotic surface of dental appliances, rather than on the mucosa. Analysis of the quantitative data shows that the number of colonies differs by time and by patient. However, in our research, we found no statistically significant differences in the number of *Candida* sp. during the 12 weeks of study. Despite this, our results suggest a positive correlation between the average number of colonies and the duration of study. The amount of oral *Candida* may fluctuate, and the lack of statistical significance may be due to the hygiene habits of patients, the food they consume, yeast biology, the small research sample, or high initial number of oral *Candida*-carriers.

Our findings regarding the upward trend of yeast growth during orthodontic treatment are consistent with the data presented in the literature [16–19]. The study of Zheng et al. [16] showed that the CFU/ml increases in users of fixed orthodontic appliances after 2 months of treatment. Lee et al. [19], in their long-term research (involving almost 12 months of observation), showed that the amount of *Candida albicans* increases after bracket bonding and peaks in the fifth month of treatment, before slightly decreasing and approaching the maximum again at the end of the first year of treatment. Arslan et al. [17], on the basis of 1 year of observation of a group of orthodontic patients, stated that a statistically significant increase in the *Candida* population occurs in the first month of orthodontic treatment. A decrease in the amount of *Candida* DNA in wash mouth samples was only found in Bergamo et al. [20] (*N* = 15, mean age 17.53 ± 8.0 years), who employed 90 days of observation after bracket application; this study group was heterogenous, consisting of 14 women and a single male. Unfortunately, the authors of none of these studies described patient preparation procedures, such as toothbrushing. Only Zheng et al. [16] stated that patients had had breakfast and had undergone their regular morning oral hygiene routine. Our recommendations were typical of laboratory tests: patients reported on an empty stomach, without having brushed their teeth. This is one of the methodological variable factors that may greatly affect results of the study.

The species profile obtained in our study is similar to that of Hägg and al [18]. and of Lee at al [19].: *Candida albicans* dominates and *C. guilliermondii* and *C. tropicalis* were isolated in small percentage*.* This may serve as a guideline for choosing antibiotic therapy for patients affected by oral mycosis. An interesting phenomenon, not noted by other authors, was the change in colonizing species seen in two patients over the course of the study (in K.J., *C. albicans* was replaced by *C. tropicalis*; and in D.M., strains of *C. guilliermondii* led to high biofilm formation; see Fig. 4). Further research may be needed to better understand the intraspecies relationships of *Candida* yeast.

Conventional orthodontic brackets have many retention areas that impede proper oral hygiene, thus leading to greater plaque build-up. In our study, the mean number of *Candida* CFUs had a tendency to grow. However, the API and GBI did not show significant changes. Interestingly, statistically significant fungal growth was seen in patients whose API decreased (indicating that oral hygiene improved) during subsequent visits, whereas patients with increasing GBI (periodontal inflammation) had lower yeast carriage. This leads to the conclusion that *Candida* sp. proliferated less in patients with an altered periodontium i.e., these were probably more colonized by bacteria. Similarly, no differences in plaque index (PI), gingival index (GI), or gingival bleeding index (GBI) during the use of fixed orthodontic appliances were observed by Bergamo et al. [20]. However, Hägg et al. [18] found a statistically significant increase in PI during the second and third visits after beginning orthodontic treatment with brackets.

One of the factors in *Candida* virulence is the ability of these yeasts to form biofilm on abiotic surfaces, including components of orthodontic appliances. The formation of a biofilm protects the cells forming it against the adverse effects of the environment, including antimicrobial drugs. Few studies have taken up the topic of the multiplication of microorganisms on orthodontic elastomers [5, 21]. Casaccica et al. [4] showed that the elastomers used in orthodontics are manufactured with due care and do not pose a biological threat. SEM images of the topography of uninfected elastic ligatures presented in this publication also confirm the absence of microorganisms. The surface of elastic ligatures however enables the formation of architecturally complex and species-rich biofilms, and is rapidly colonized during orthodontic treatment.

It would seem that strains isolated from patients colonized while using orthodontic appliances will form a biofilm well. For our patients, the level of colonization before treatment was high, and during the course of treatment, the ability to create biofilm did not change, except in sporadic cases. This indicates that, while the number of yeasts including those derived from biofilms produced in the oral cavity increased, the biofilm-forming potential (virulence) of the isolated strains did not change. It can thus be concluded that, while oral appliances promote colonization by yeasts, they do not increase their virulence. Appropriate antifungal prophylaxis should thus permit control of the development of oral mycosis during long-term orthodontic therapy. However, in vivo biofilm formation may not coincide with in vitro biofilm formation capacity. The model does not take into account all factors (especially the presence of bacteria). The *Candida* pathogenicity (biofilm formation) described in the literature thus does not coincide with the pathogenicity of strains in vivo.

## Conclusions

- Treatment with orthodontic appliances promotes colonization by *Candida* yeast.
- Yeast colonization is variable over time in terms of strain and species of fungi, with the dominance of *Candida albicans*.
- In patients who are carriers, the API value decreases over time, and in uncolonized patients the GBI value increases; this may have predictive significance for the development of oral candidiasis during orthodontic treatment, but further research is required to confirm these relationships and determine the cut-off point.
- Strains isolated from orthodontically treated patients do not show increased biofilm-forming activity.

## Data Availability

The datasets used and analyzed in this study are available from the corresponding author on reasonable request.

## Abbreviations

API: Approximal plaque index
GBI: Gingival bleeding index
SD: Standard deviation
CFU: Colony-forming unit
RPMI: Roswell Park Memorial Institute
MOPS: 3-morpholinopropane-1-sulfonic acid
PBS: Phosphate buffered saline
PI: Plaque index
GI: Gingival index
ATCC: American-Type Culture Collection
